# Additive Manufacturing of Sub-Micron to Sub-mm Metal Structures with Hollow AFM Cantilevers

**DOI:** 10.3390/mi11010006

**Published:** 2019-12-18

**Authors:** Giorgio Ercolano, Cathelijn van Nisselroy, Thibaut Merle, János Vörös, Dmitry Momotenko, Wabe W. Koelmans, Tomaso Zambelli

**Affiliations:** 1 Exaddon AG, Sägereistrasse 25, 8152 Glattbrugg, Switzerland; thibaut.merle@exaddon.com (T.M.); wabe.koelmans@exaddon.com (W.W.K.); 2 Laboratory of Biosensors and Bioelectronics, ETH Zürich, Gloriastrasse 35, 8092 Zurich, Switzerland; vannisselroy@biomed.ee.ethz.ch (C.v.N.); janos.voros@biomed.ee.ethz.ch (J.V.); momotenko@biomed.ee.ethz.ch (D.M.)

**Keywords:** additive micromanufacturing, electrochemical microprinting, copper microcoils, FluidFM, David

## Abstract

We describe our force-controlled 3D printing method for layer-by-layer additive micromanufacturing (µAM) of metal microstructures. Hollow atomic force microscopy cantilevers are utilized to locally dispense metal ions in a standard 3-electrode electrochemical cell, enabling a confined electroplating reaction. The deflection feedback signal enables the live monitoring of the voxel growth and the consequent automation of the printing protocol in a layer-by-layer fashion for the fabrication of arbitrary-shaped geometries. In a second step, we investigated the effect of the free parameters (aperture diameter, applied pressure, and applied plating potential) on the voxel size, which enabled us to tune the voxel dimensions on-the-fly, as well as to produce objects spanning at least two orders of magnitude in each direction. As a concrete example, we printed two different replicas of Michelangelo’s David. Copper was used as metal, but the process can in principle be extended to all metals that are macroscopically electroplated in a standard way.

## 1. Metal Additive Manufacturing at the Micro Scale

At the macro- and mesoscale (down to 100 µm), additive manufacturing (AM) of metal objects is recurrently performed with robust methods like selective laser and electron beam melting, both relying on the local fusion of metal particles to obtain a solid metal with the desired shape [1,2,3,4,5]. Typical voxel sizes are reported in the range of 100 to 400 µm [6] As alternatives, one can take into consideration the well-established localized electrochemical deposition (LED) [7,8] and laser chemical vapor deposition (LCVD) [9] methods, whose minimum feature sizes are continuously improved down to 10 µm [10,11] and correspondingly interpreted [12,13]. Restricting the depiction only to the fabrication of metal objects at the micron scale (i.e., with details smaller than 10 µm, µAM), three main strategies are being established [14]: fabrication of templates by micro-stereolithography to be successively metallized either by coating (positive templates) or by electroplating (negative templates) [15,16,17,18], transfer of metallic materials to be eventually sintered in a second step, or in situ metal synthesis. As an example of the “transfer” strategy, we can refer to direct ink writing [19,20,21], electrohydrodynamic printing [22,23,24], laser-assisted electrophoretic deposition [25], laser-induced forward transfer [26], melt droplets [27]. On the other hand, focused ion/electron beam methods (metal precursor dissociation) [28,29,30], as well as laser-induced photoreduction [31,32,33], together with electrochemical reduction (meniscus-confined) [34,35], local dispensing [36,37], electrohydrodynamic redox printing [38], can be catalogued in the “in situ metal synthesis” ensemble. More recently, other methods have appeared like pyrolysis of metal-containing resins structured with two-photon lithography [39], as well as implosion fabrication [40].

*Electrochemical additive manufacturing at the micro scale.* Since in this contribution we are presenting results obtained via local electrochemical (ec) reduction, we would like to focus on the techniques of this category. Their common underlying idea is to fill a printing nozzle (in most cases a pipette) with a solution containing a metal salt, to bring it as close as possible to a biased conductive substrate (generally connected as working electrode, WE), and to eject the solution inducing a confined metal plating because of the vicinity of the nozzle with the working electrode. Based on this idea, four different protocols have been developed; meniscus-confined electroplating, scanning ion conductance electroplating, electrohydrodynamic redox printing, force-controlled electroplating.

In the case of meniscus-confined electroplating (MCEP), which is performed in an atmosphere with controlled humidity, a glass pipette containing the metal ion solution and equipped with a counter electrode (CE) is moved toward the WE until a meniscus is formed between them. Inside the meniscus, the ec cell reduction takes place, forming a narrowed metal deposit that can be interpreted as a “voxel” [34]. In this way, metal wires were produced [41], eventually branching out in 3D structures [35]. The wires obtained show a diameter even smaller than the aperture diameter which is typically in the range between 100 and 250 nm. As the metals, copper and platinum were electroplated. Recent MCEP developments are focusing on implementing a feedback for the pipette approach by taking advantage of the ec current flowing once that the meniscus is established [42], as well as on the parallelization to enlarge the printed area by utilizing nozzle arrays [43,44].

For scanning ion conductance electroplating (SICM-EP), a double-barrel glass capillary is immersed in an ec cell, each barrel containing a CE. One barrel is filled with the copper salt solution, whereas the other one with a salt solution (i.e., same as in the bulk of electrolyte and without any metal ions). This second barrel without metal ions is responsible for the SICM feedback during the approach toward the WE, while the first one serves as source of metal ions to be reduced in the volume between pipette apex and WE [37]. High aspect-ratio structures, together with overhanging ones, could be manufactured, whose section was found to be 10–20 times larger than that of the aperture (30–50 nm). In a recent publication [45], Yoshioka and coauthors utilized a similar double-channel pipette configuration, whereby one channel was assigned to the SICM feedback for the apex-substrate separation, while the other was filled with a gold colloidal solution (3 nm) for electrophoretic deposition of the Au nanoparticles, obtaining high-aspect ratio micropillars with diameter in the sub-micron range (~800 nm diameter with a nozzle aperture of ~200 nm).

Electrohydrodynamic redox printing (EHD-RP) also uses double-barrel capillary glasses, each barrel containing solvated metal ions generated via in situ ec dissolution of a metal electrode (copper and silver) [38]. Ion-loaded solvent droplets are then expelled by electrohydrodynamic forces and reduced to a metal deposit on the WE (~400 nm diameter with a nozzle aperture of ~200 nm). The biggest novelty of this method is inherent to the possibility of manufacturing multimaterial microstructures.

**Figure 1:**
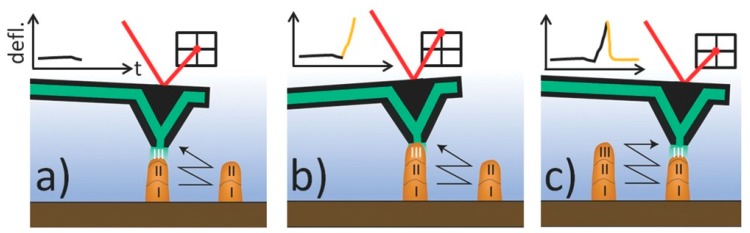
Automated force-controlled electrochemical (ec) 3D printing process schematized [36] the simple case of two pillars side-by-side: we emphasize the layer-by-layer fabrication (layers I-III are labeled), i.e., the pillars are printed in parallel and not in series. (**a**) The ion tip filled with CuSO_4_ solution is positioned over the first pillar at a set separation (e.g., 500 nm) where the metal voxel is to be deposited. Local electroplating is switched on at a given overpressure, leading to local pillar growth (voxel III). (**b**) When the growing voxel touches the pyramidal apex, a cantilever deflection is detected on the photodiode via the moving laser beam. The inset graph shows the temporal evolution of the deflection signal (defl.) for a voxel touching event (yellow segment). (**c**) As soon as this touching event is recognized by the software, the probe is moved to the next position, i.e., on top of the second pillar again with a typical separation of 500 nm, and the voxel ec deposition is started. Reprinted with permission from Ref. [36]. Copyright 2017 John Wiley & Sons Inc.

*Force-controlled electroplating (FCEP).* At the core of the method lies the FluidFM technology [46,47], which combines atomic force microscopy ([48], AFM) and microfluidics by means of AFM cantilevers with an embedded microchannel and an aperture at their apex. When used for µAM, these hollow AFM probes are termed *ion tips* (Exaddon AG, Glattbrugg, Switzerland). Upon immersion in a standard three-electrode ec cell, the ion tip could be used as nozzle for local supply of precursor ions (copper), thus confining their ec reduction on the cathodically polarized WE [49]. This elicited a novel protocol for 3D µAM of metal structures [36]. By setting an initial separation of a few hundred nanometers between the probe aperture and the substrate, the locally plated metal can freely grow in the vertical direction, creating a voxel and thus giving access to printing in the third dimension. Moreover, the protocol could be automated by relying on the force-sensing capability: by detecting the moment when the growing voxel touches the probe apex, the deflection signal can be used as a trigger to define when the voxel deposition has ended. Subsequently, the probe is moved to the next coordinate where the following voxel is planned to be deposited. Therefore, complex microstructures could be fabricated in a true layer-by-layer fashion (Figure 1).

As metal, copper is mainly chosen for ec printing because of its coulombic efficiency (minimum faradaic losses due to side reactions) beside its importance for electronic applications. In a recent comprehensive study, the mechanically properties were compared for two benchmark structures produced with most methods mentioned in this introduction [50].

We conceived this contribution to describe the FCEP in detail revisiting our two main publications [36,51] but also presenting complementary unpublished results.

## 2. The Force-Controlled Microprinting Tool

**Figure 2:**
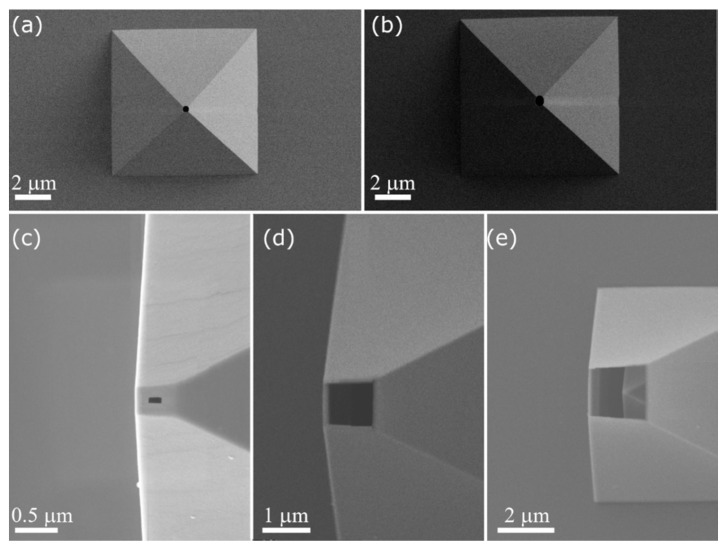
Scanning electron microscope (SEM) micrographs of the apertures at the apex of the pyramid of the ion tips. Top view of a (**a**) 300 nm and a (**b**) 500 nm diameter aperture, obtained by contact lithography. Top view of a (**c**) 100 nm, a (**d**) 1 µm and a (**e**) 2 µm side square aperture, obtained by focused ion beam (FIB) milling of closed pyramidal probes. The choice of the smallest aperture dimension of 100 nm was conditioned to avoid potential complications in filling the probes with electrolyte [54], whereas 2 µm was the maximum size possible due to the limit set by the presence of the apex of the inner pyramid (discernable in (**e**)). Reprinted with permission from Ref. [51]. Copyright 2019 John Wiley & Sons Inc.

*Microchanneled AFM probes.* AFM cantilevers were produced with an embedded microchannel connecting the hollow pyramidal tip on one side and a macro reservoir on the other side (ion tips, Exaddon AG). Such microchannels were obtained either by etching a sacrificial polysilicon layer between two layers of Si_3_N_4_ according to the batch processes reported in [52,53]. The standard section of the microchannel is of 20 µm × 1 µm, whereas the pyramid is 10 µm × 10 µm × 7 µm with a circular aperture at the apex either of 300 nm or of 500 nm diameter (Figure 2). Alternatively, for apex apertures of different sizes, we opened so called “closed” probes (i.e., having no aperture at the apex) by means of a focused ion beam (FIB) milling. In this case, the probes were mounted in a custom probe holder and coated with an 18-nm-thick carbon layer using a CCU-010 Carbon Coater (Safematic GmbH, Bad Ragaz, Switzerland) before milling by a FIB-scanning electron microscope (SEM) Nvision 40 device (Zeiss, Oberkochen, Germany) with the SmartSEM software (Zeiss). The milled face of the pyramidal probe was aligned in parallel with the FIB-beam equipped with a gallium ion source. Subsequently, the probes were milled with an acceleration voltage of 30 kV at milling currents varying from 10 pA up to 80 pA, depending on the desired opening. The active milling process was followed in live SEM mode and was only terminated when the desired opening size was reached. After the milling process, the probes were glued onto a dedicated printing holder (Exaddon AG). AFM approaches were always performed in contact mode in the standard optical beam deflection method.

*ec cell.* The first version of the electrochemical cell consisted of gold 15-nm-thin films on glass with 3 nm Ti adhesion layer as WE, an Ag wire as quasi-reference electrode (RE), and a Pt wire as CE in a simple droplet of H_2_SO_4_ at pH 3 [36]. The second version of the printing chamber (i.e., the three-electrode cell) was redesigned in a modular fashion with the parts made out of solid Teflon [51]. The working electrode is connected to the printing substrate of 12 mm × 12 mm; furthermore, a graphite counter electrode and a Ag/AgCl wire are used as reference. As WE, 100-nm-thick films of sputtered Cu on a 1.3 cm × 1.3 cm silicon substrate with 13 nm of Ti adhesion layer were used with a graphite CE and a Ag/AgCl wire as RE.

The supporting electrolyte was a 0.5 M H_2_SO_4_ with the addition of HCl to a concentration of 0.5 mM. The plating solution was a 0.8 M CuSO_4_ in 0.5 M H_2_SO_4_ solution.

**Figure 3:**
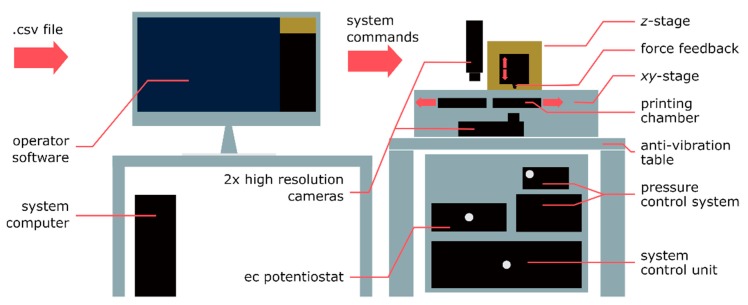
Schematic of the force-controlled ec µAM system. On the left, a system computer sends commands to the system control unit on the right. The system control unit governs the printing process using an embedded controller. The ion tip is mounted on the z-stage in the printing head and is moved inside the ec deposition cell by the z-and x-y stages. A microfluidics control system with 1 mbar precision regulates the electrolyte flow through the cantilever aperture. Adapted with permission from Ref. [51]. Copyright 2019 John Wiley & Sons Inc.

*Setup*. The prototype printer was obtained assembling an AFM head (Nanowizard I by JPK, Berlin, Germany), a pressure controller (MFCS-4C by Fluigent, Villejuif, France), and a potentiostat (PalmSens, Utrecht, The Netherlands) to be synchronized together [36]. Automation was achieved by a custom-made LabVIEW program which controlled the probe position and read the deflection signal from the AFM via two data acquisition cards (NI-USB 6009 and NI-USB 6343, National Instruments, Ennetbaden, Switzerland). However, for sizes in the z direction exceeding the piezo range of 15 μm, further z increments could only be accomplished with the AFM stepper motors, whereby their operation introduced x-y offsets which had to be accounted for by printing an additional pillar close to each structure. The tip end of this continually increasing reference pillar had to be found after each stepper movement by automatically performing a rough AFM scan in the pillar area at a z value corresponding to the height of the current pillar. The x-y error introduced was then determined and successfully compensated, but at the cost of important time delays. To get rid of this deficiency, the printing tool was completely redesigned [51]. Here, we introduce a completely redesigned system as far as both the hardware and the software are concerned. A controller (Exaddon AG) was conceived with a low latency and a fast feedback loop in order to react to a touching event (Figure 3) within 1 ms. In this way, possible clogging of the opening is minimized, thus increasing the probe lifetime while higher printing rates are also permitted. On the other side, the controller buffer is able to contain the next 16 voxel coordinates to avoid any delay by a lack of voxel coordinates in the controller for stable and long-term operation up to tens of hours. Further components of the hardware of the new system are two high-resolution optical systems (top and bottom view) for loading of the nozzle, for printer adjustment and calibration, as well as for imaging of the printed structures. The maximum volume of the printing space is 200 mm × 70 mm × 60 mm, assured by a three-axis positioning stage (Exaddon AG) consisting of three linear motors with an x-y positioning accuracy (full-range, repeatability) of 250 nm, as well as with 5 nm positioning accuracy in *z* at a 0.1 nm sensor resolution. The corresponding repositioning time from a voxel to the following one takes place in less than 50 ms (for a *Δz* of 500 nm).

## 3. Tuning the Voxel Size at Different Scales: from 0.5 to 20 µm

The ec µAM system is a multiparameter instrument, whereby the value of the probe aperture *D*_eq_, of the applied pressure *p*, of the electrodeposition potential *E* as well as the voxel height *Δz* can be precisely adjusted.

**Figure 4:**
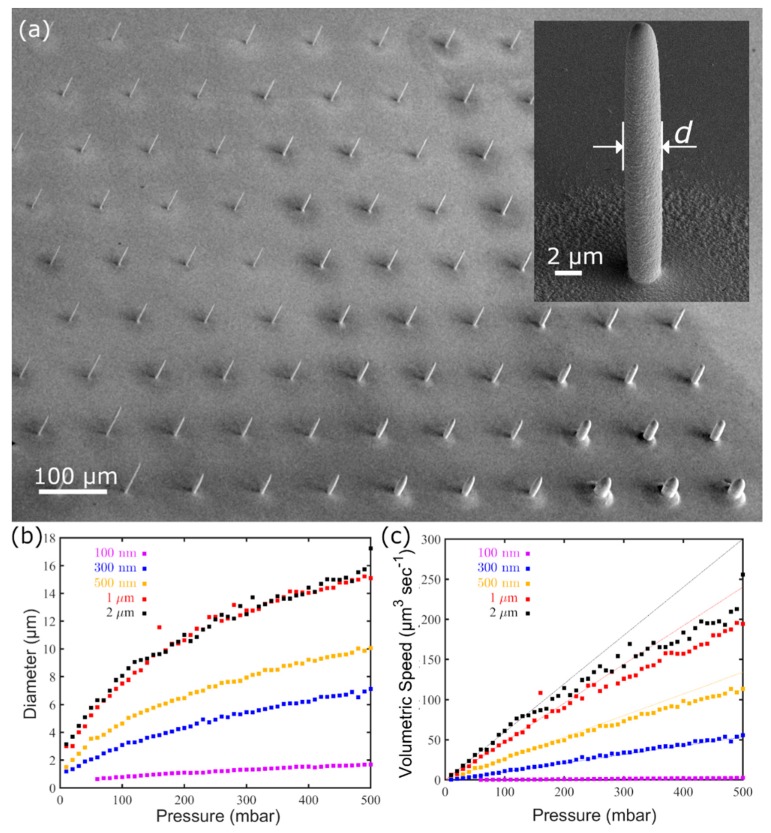
(**a**) SEM image of a field of pillars printed at different pressure and deposition potential values with a 300 nm aperture ion tip, while a SEM image of a single pillar with overlaid the results of the diameter d analysis is shown in the inset. (**b**) Pillar diameters d obtained as a function of the pressure applied during printing and, (**c**) volumetric deposition speed $\dot{z}$ as a function of the pressure. Panels (**b**) and (**c**) reprinted with permission from Ref. [51]. Copyright 2019 John Wiley & Sons Inc.

In a previous work [51], we investigated the effect of the ion tip aperture *D*_eq_ and *p* on the diameter *d* of a pillar printed at fixed potential (E = −0.5 V vs. Ag-AgCl) and geometry (i.e., distance between two consecutive voxels *Δz*, here referred to as voxel height) as in Figure 4a. The framework consists in measuring the pillar diameter d from SEM images (Figure 4a, all the *d* values are compiled in Figure 4b) and the vertical speed $\dot{z}$ from the printer logged z-axis position. These two experimental values can then be combined to calculate the volumetric deposition speed $\dot{V}$:

(1) $$\dot{V}=\frac{\pi }{4}d^{2}\dot{z}$$

as function of opening size *d* and *p* (Figure 4c).

In this work, the same protocol is used to investigate the effect of the deposition potential *E*.

Arrays of pillars were printed at pressures varying from 10 mbar to 210 mbar with a pressure step of 5 mbar. Each array was printed at a different deposition potential, the potential values were changed from −0.5 V to −0.42 V with a 20 mV difference between each array; the deposition potential was set against an Ag/AgCl pellet as RE and all the given potential values are referred to in this reference.

**Figure 5:**
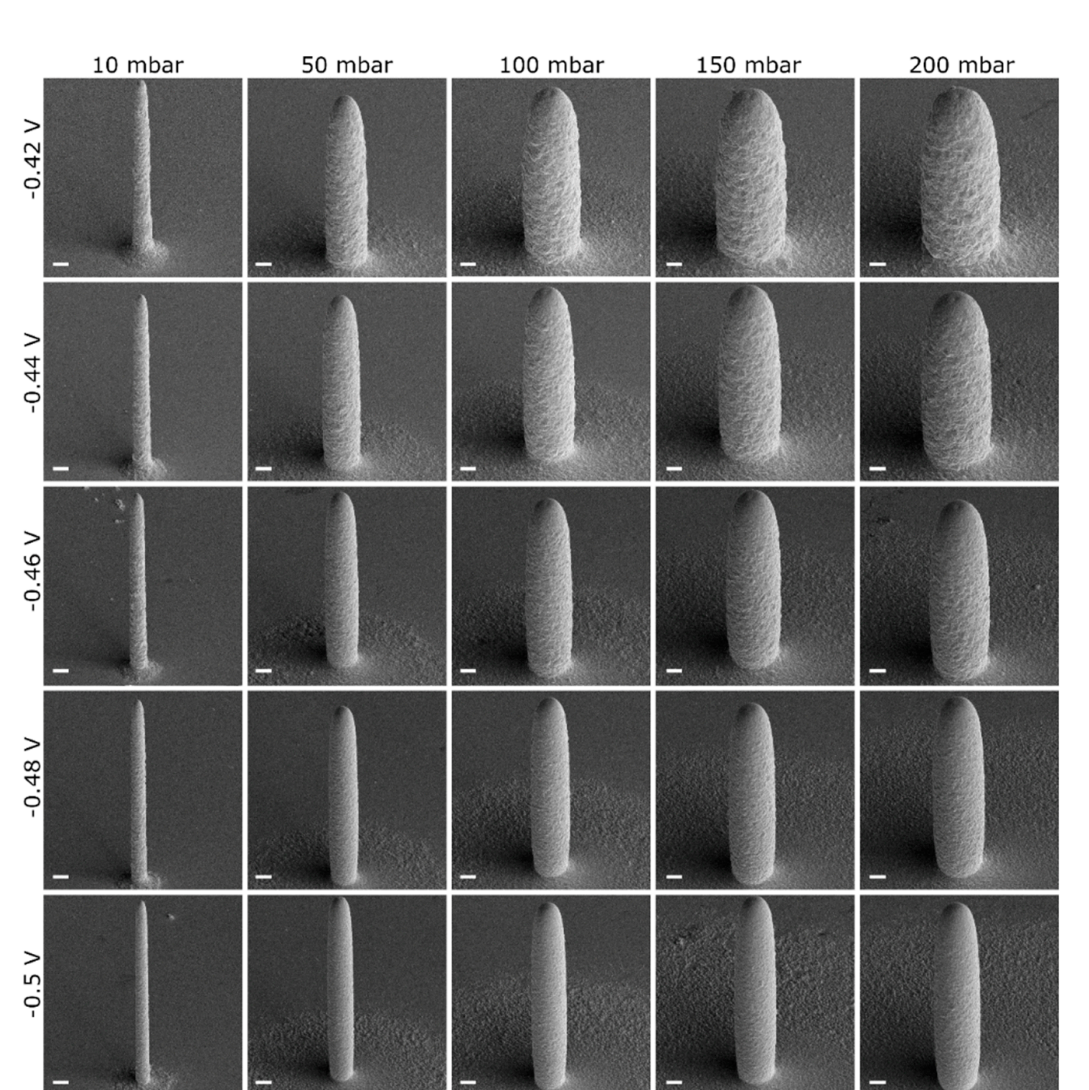
SEM images of pillars deposited with a nozzle aperture of 300 nm with 10, 50, 100, 150 and 200 mbar applied pressure and −0.42, −0.44, −0.46, −0.48 and −0.50 V deposition potential. All the images are taken at the same magnification (the scale bar corresponds to 2 µm).

Figure 5 shows a set of selected SEM images showing pillars printed at selected pressure values. In agreement with [51], increasing *p* while keeping *E* constant results in an increase of the diameter *d*. A larger flow of ions requires a larger area for the ions contained in it to be completely consumed by the reduction reaction so that the pillars grow with a wider cross-section. By contrast, an increase in *E* (i.e., more negative deposition potential) has an opposite effect: pillars printed at the same pressure but at increasing *E* have a smaller cross-section.

This result highlights that electrochemical printing is fully controlled by the electrodeposition potential and pressure, while it is monitored by measuring the retraction speed of the nozzle, which is automated with the force feedback. In principle, the arrangement of the ec cell resembles the so-called wall-jet electrode system, a tool in hydrodynamic voltammetry that utilizes a jet of solution of electroactive species, which is pushed from a circular nozzle to hit the working (collector) electrode perpendicularly [55]. The diffusion limited current *I_d_* is well-described in the wall-jet arrangement and is approximated by analytical expressions that relate it to the geometrical parameters of the system (nozzle, *a*, and working electrode radius, *R*) and the parameters of the fluid flow:

(2) $$I_{d}=1.6\;k\;n\;F\;c_{0}\;D^{2/3}\;\upsilon^{-5/12}\;d^{-1/2}R^{3/4}\;V^{3/4}$$

where *k*, *n*, *F*, *c*_0_, *D*, *ν* and *V* specify geometrical proportionality factor, number of electrons in ec reaction, Faraday constant, concentration of electroactive species, their diffusion coefficient, dynamic viscosity of the medium and volumetric flow rate, respectively. This expression can be simplified by arranging all the constants into a proportionality factor α (different for each voltage value):

(3) $$I=\alpha R^{3/4}\;p^{3/4}$$

**Figure 6:**
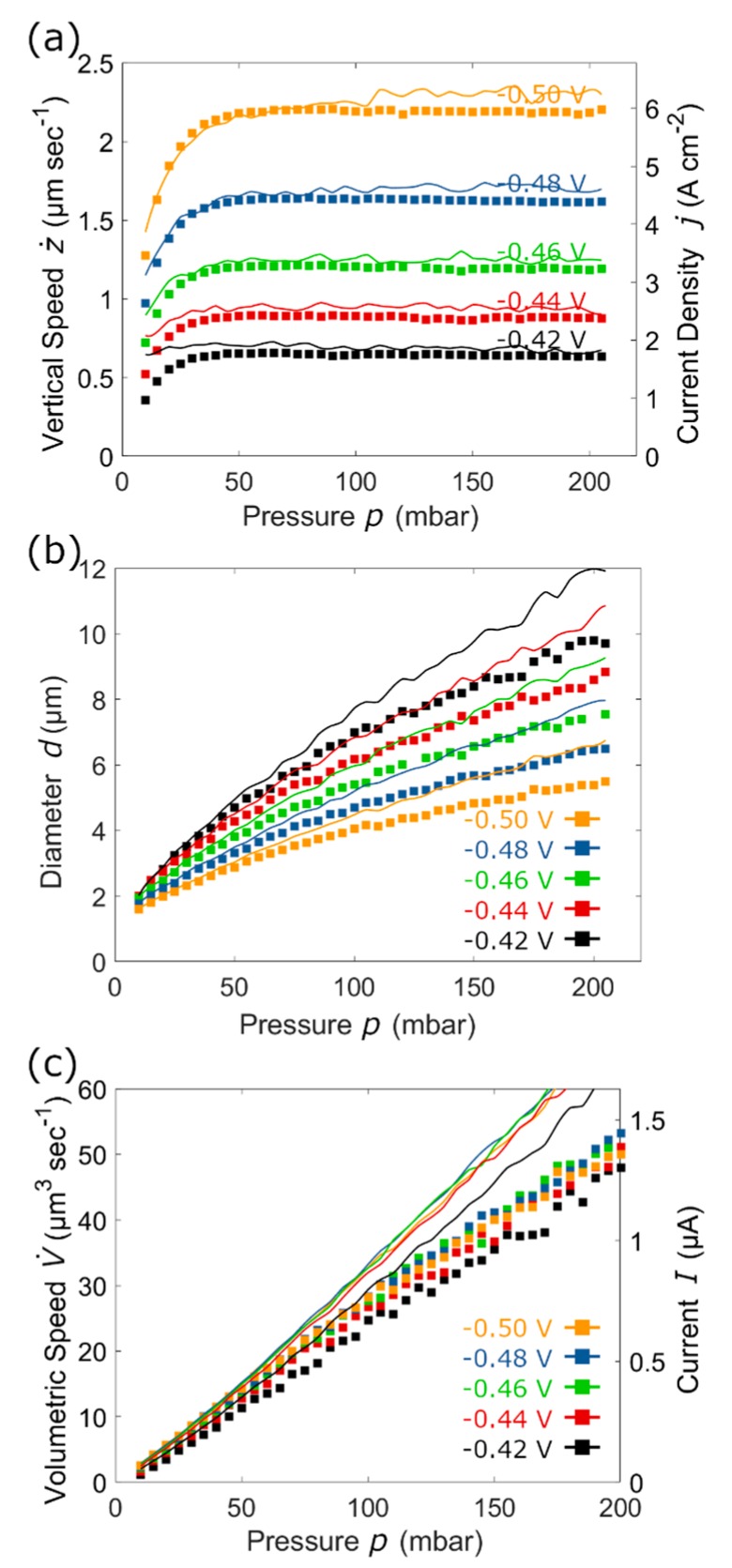
(**a**) Vertical plating speeds (dots), (**b**) pillar diameters and, (**c**) volumetric deposition speeds as a function of the pressure applied during printing at different deposition potentials. Dots are the experimental data, whereas lines the fitted data. Because of the semi-empirical fitting, the lines have a characteristic shape.

The deposition current could not be measured directly because the active Cu^2+^ reducing area is in the order of tens of µm^2^, while the total working electrode area is cm^2^, resulting in the deposition current being completely masked by the large background current on the working electrode. However, using the vertical growth speed (Figure 6a) along with experimentally measured pillar diameters (Figure 6b), it becomes possible to derive further insights of the printing process. For example, the vertical copper growth rate (using the given Cu molar mass and density, *M*_Cu_ and *ρ*_Cu_), can be estimated from the Equation (3) by a simple transformation:

(4) $$V=\alpha \frac{M_{\mathrm{Cu}}}{\pi \rho_{\mathrm{Cu}}}R^{-5/4}\;p^{3/4}$$

This relationship is complicated by the fact that the radius of the growing pillar *R*, which collects metal ions upon electroplating, is in fact a function of the applied pressure *R* = *R*(*p*) (Figure 6b). For better fitting of the vertical growth data using the experimental values of pillar diameters *R*_experiment_(*p*), we introduced an empirical scaling coefficient that allows estimation of the radius of the area where ions are collected *R*_fit_(*p*):

(5) $$R_{\mathrm{fit}}\left(p\right)=e^{p/1000}\;R_{\mathrm{experiment}}\left(p\right)$$

The result of this estimation suggests that the area where the current is collected upon plating at higher pressures (*p* > 60 mbar) is somehow larger than the printed metal pillar. Volumetric deposition rate $\dot{V}$ also deviates from the theoretical prediction, highlighting that at higher pressures some portion of the metal ions is electroplated around and not directly on the pillar. These results are in good agreement with electron microscopy images (Figure 5) that depict circular «dunes» around the pillars, with sizes increasing at larger pressure magnitudes.

## 4. Arbitrary-Shaped 3D Structures in a Layer-by-Layer Fashion: Michelangelo’s David

The confined electrodeposition is not limited at strand printing of pillars and coils. The same system can also be using to produce complex 3D object by taking advantage of the merging of voxels placed side by side. To demonstrate the capabilities of this process, a 3D model of Michelangelo’s David (author of the original model ‘gabrielmda’, distributed under CC-0 license on blendswap.com) was modified and sliced using the open-source 3D modeling software Blender 2.8 (Blender Foundation, Amsterdam, the Netherlands). The voxels defining the structure were routed in a layer-by-layer fashion using a custom clustering algorithm. The same model was then reproduced at a 10,000:1 and a 70,000:1 scale.

**Figure 7:**
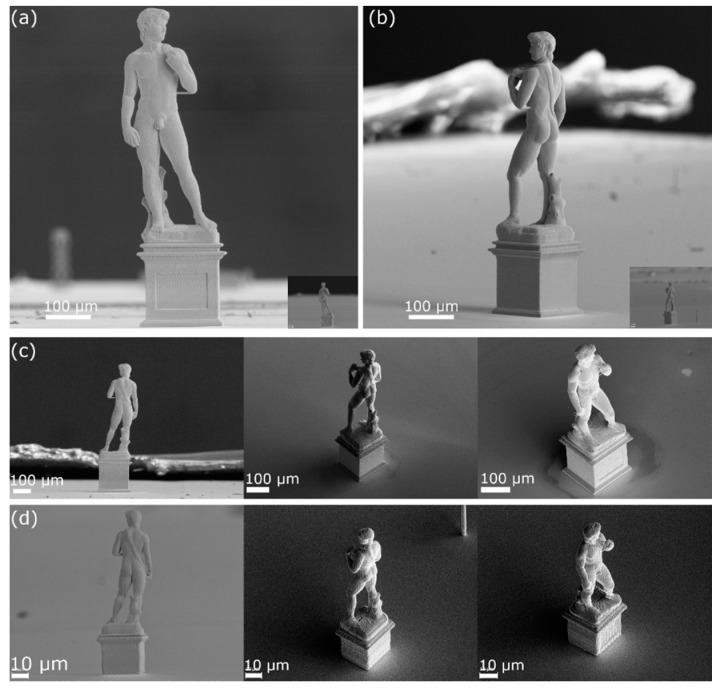
SEM images of a 1:10,000 and a 1:70,000 replica of the David (Michelangelo). (**a**), (**b**) and (**c**) are the 1:10,000 scaled, 700 µm-tall replica imaged from different angles, the insets in (**a**) and (**b**), and the panel (**d**) are the 1:70,000 scaled, 100 µm-tall replicas.

The two replicas of Michelangelo’s David [56], at the two completely different scales shown in Figure 7, represent the current state-of-the-art in confined electrodeposition 3D printing. The 700 µm-tall 1:10,000 replica is shown in Figure 7a–c; the structure is defined by 130,000 voxels and the total print time required is 16 h using a pressure of 50 mbar and a deposition potential of −0.46 V. The 100-µm-tall, 1:70,000 version of the same model is shown in Figure 7a,b inset (to allow a direct comparison between the two objects) and 7d; this structure is defined by 25,000 voxels and the printing time required is 2 h using a pressure of 15 mbar and a potential of −0.5 V.

The two different pressure and potential values were utilized to optimize the print time and resolution required: the larger pressure and lower deposition potential used on the 700 µm structure allowed for the definition of a geometry with larger voxels (~4 µm diameter, as from Figure 6b), thereby reducing the total voxel number and print time required, while keeping the resolution to an acceptable value for the scale of the object. By contrast, for the smaller 100 µm replica a higher resolution was required to target smaller voxels (~1.6 µm diameter, as from Figure 6b), therefore lower pressure and higher overpotential were employed.

## 5. Intertwined Coils with Different Section Sizes

**Figure 8:**
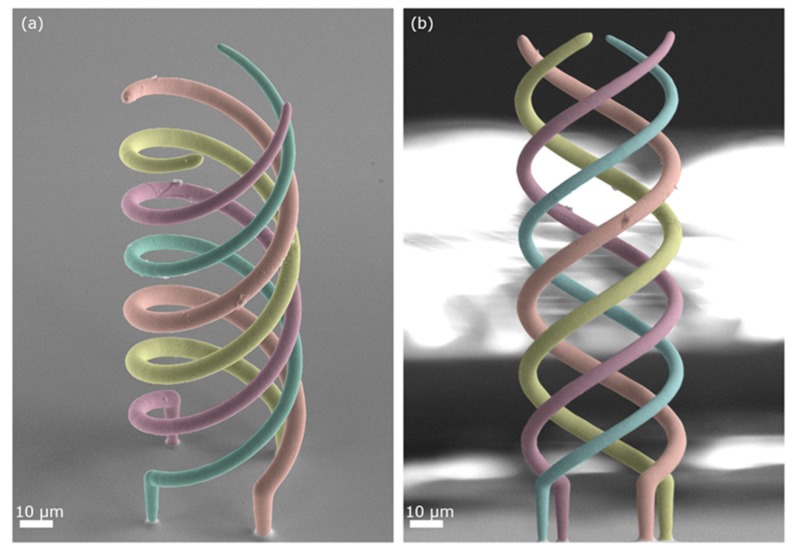
SEM images (colored) of four intertwined coils printed with a 500 nm nozzle, each coil printed at a different pressure to highlight the flexibility and the ability to change and control the voxel size within the same structure during the printing process; 3056 voxels were printed in 25 min to obtain the 180 µm tall structures. (**a**) and (**b**) are two different views of the same object. Reprinted with permission from Ref. [51]. Copyright 2019 John Wiley & Sons Inc.

Herein we also demonstrate how it is possible to tune the voxel area by adjusting *p* on-the-fly to fabricate a structure composed of parts with different sections. For this purpose, we designed a structure of four intertwined coils, each wire having its own diameter. If fabricated with a single nozzle, such an entangled object can be produced only in a layer-by-layer fashion, on the condition that each of the four voxels of each layer is plated at a different value of pressure. A probe with a 500 nm aperture was operated at 20, 40, 60, and 80 mbar in succession (Figure 4b) obtaining the structure presented in Figure 8, whereby wires are presented, artificially colored for better visualization.

The main difficulty of growing multiple threads in parallel each at different pressure values is the time response required to establish a stable pressure; specifically, whether or not the pressure and thus the cloud of Cu^2+^ ions can change quickly enough when the nozzle is moved from one strand to the next (which takes just a few milliseconds). As evincible from Figure 8, each printed wire has indeed a different diameter, which is homogeneous along all its length (5, 6, 7 and 8 µm, respectively). From Figure 4b it would be expected that the diameters at those particular pressure values are 2, 3, 3.7 and, 4.4 µm. Experimentally, the diameters of the strands of the intertwined helices are consistently larger than those measured for the individual pillar. This bigger voxel size is probably due to the “additional” plating taking place during the lateral motions from a strand to the next. Such lateral motions are absent in the printing of pillars which occurs only in the z direction.

## 6. Conclusions and Outlook

In conclusion, we have introduced a new one-step method to do additive micromanufacturing of metallic structures using microchanneled AFM probes. The key novelty of this approach is that the voxel growth can be detected in situ by monitoring the real-time deflection of the hollow cantilever. This is essential to avoid clogging of the probe aperture and to automate the process. Furthermore, it removes the need to calibrate parameters such as growth speed. Finally, the fact that the manufacturing takes place in a supporting electrolyte allows for the reliable voxel-wise printing on arbitrary positions without the restrictions of methods relying on meniscus formation. Combining these advantages, an ec 3D metal printing is achieved in a true voxel-by-voxel layer-by-layer fashion, enabling the fabrication of arbitrarily shaped geometries. This is a breakthrough in metal 3D printing on the micrometer scale, enabling a new range of printable shapes and structures.

In a second step, the force-controlled ec µAM was completely redesigned to optimize the ec printing of copper by reducing the response and positioning times and increasing the motor range. We gained further understanding of the deposition process by investigating the pillar growth systematically varying the ec deposition potential, the applied pressure and the probe aperture at a fixed the voxel height (*Δz* = 500 nm). The influence of the applied pressure and the probe aperture on the lateral diameter of the electroplated copper voxels was also investigated and rationalized. In this way, the voxel area could be spanned over two orders of magnitude using the same probe aperture. This multi-scale capability was exploited to fabricate copper microstructures of two different replicas of Michelangelo’s David. Lastly, a 50 µm × 50 × µm 170 µm object consisting of four helical wires was obtained by changing the pressure on a voxel-by-voxel basis so that each printed wire had a different diameter, yet homogeneous along its entire length. A significant throughput increase is now imaginable with the possibility of tuning the voxel size depending on the feature dimensions within the same design. Copper is the most compliant metal for electrodeposition, nonetheless this protocol can be applied in a one-to-one way to other metals.
